# Prevalence of Bandemia in Respiratory Viral Infections: A Pediatric Emergency Room Experience

**DOI:** 10.3389/fped.2020.576676

**Published:** 2020-12-29

**Authors:** Estela Noyola, Asif Noor, Nicole Sweeney, Joshua Chan, Rahul Ramesh, Rose Calixte, Leonard R. Krilov

**Affiliations:** ^1^Department of Pediatrics, NYU Long Island School of Medicine, Mineola, NY, United States; ^2^Department of Community Health and Social Medicine, The City University of New York (CUNY) School of Medicine, New York, NY, United States

**Keywords:** bandemia, respiratory viral infections, fever, complete blood cell (CBC) count, absolute neutrophil count (ANC), absolute band count, film array

## Abstract

**Objective:** The aim of this study was to examine the prevalence of bandemia in confirmed respiratory viral infections in febrile infants and children presenting to the emergency department.

**Methods:** An observational retrospective study from January 1, 2016, through December 31, 2016, was conducted in patients between the ages of ≥ 1 month and ≤ 5 years presenting to the emergency room with fever and who had a complete blood cell count performed. Patients were separated into seven groups based on the type of respiratory viral infection. Inclusion criteria strictly counted children with viral infections and absence of clinical and laboratory evidence of a bacterial coinfection.

**Results:** A total of 419 patients had a documented viral infection. A significant proportion of these children were found to have bandemia; children with adenovirus (17%), respiratory syncytial virus (RSV) (14.9%), human metapneumovirus (hMPV) (13%), and parainfluenza virus (7.9%) had the highest prevalence when the cutoff for bandemia was set at 10%. The prevalence increased to 35.3, 30.9, 40.3, and 15.8% for adenovirus, RSV, hMPV, and parainfluenza virus, respectively, when this cutoff was lowered further to 5%.

**Conclusion:** Band neutrophils are detected frequently in confirmed respiratory viral infections particularly during early stages.

## Introduction

Respiratory viral infections, either confirmed or presumed, account for the majority of febrile illnesses in healthy infants and young children (1, 2). Common viral presentations include fever with upper respiratory signs and symptoms, clinical bronchiolitis, or fever without localizing signs (3). The diagnosis of a viral infection is mainly based on clinical grounds. The multiplex molecular respiratory panel (respiratory PCR assay) has enabled better identification of these respiratory viral infections and provides objective data. Many of these children would get non-specific laboratory markers performed, i.e., complete blood count (CBC), C-reactive protein (CRP), and erythrocyte sedimentation rate (ESR) to investigate a concomitant bacterial infection (4). Since the 1990's, there have been multiple studies and guidelines on the management of febrile infants, which indicated a low, but non-negligible, risk of serious bacterial infections (5–7). Infants > 1 month of age who present to the emergency room (ER) often get a CBC as part of the workup. This study sought to investigate the prevalence of bandemia in a cohort of children with confirmed viral infection.

## Methods

We conducted a retrospective study from January 1, 2016, through December 31, 2016, in children between the ages of ≥ 1 month and ≤ 5 years. These children presented to our Children's Medical Center, which is a suburban, tertiary-care academic hospital in the Northeast United States with 95 pediatric inpatient beds and 19,000 pediatric ED visits per year. They presented with documented fevers defined as a temperature of 100.4° Fahrenheit (38° C) or greater and had a complete blood cell count performed using Beckman Coulter ® LH 780 electric cell counter. A manual differential count of the peripheral blood smear was performed on every patient, which is standard protocol in patients 5 years or younger in our institution (**Figure 3**). Febrile children with confirmed viral infections (BioFire FilmArray® multiplex PCR) were included in the study. We excluded patients with chronic conditions (sickle cell disease, malignancy, and inflammatory bowel disease), immunodeficiencies, patients with diagnosed bacterial infections, and toxic appearing children as documented by attending physicians. We also excluded children with documented signs of acute otitis media because it is difficult to distinguish between viral or bacterial etiology. Cases where the chest x-ray was read as focal infiltrates due to atelectasis by the attending radiologists were included in the viral infection group and cases with chest x-ray findings of consolidation, effusion, or empyema were excluded to ensure that patients did not have a bacterial coinfection.

Cases with confirmed viral infections had nasopharyngeal specimens obtained and tested positive for Respiratory syncytial virus (RSV), adenovirus, influenza, parainfluenza, and human metapneumovirus (hMPV) on the Filmarray® multiplex PCR system. We excluded enterovirus and rhinovirus because of difficulty in distinguishing symptomatic infections from asymptomatic prolonged shedding. Also, results are reported together as enterovirus/rhinovirus (both are picornavirus) and it is difficult to determine which virus is the culprit. We also excluded coronavirus (non-SARS or MERS) as a majority of the cases were either asymptomatic or had mild upper respiratory symptoms.

In addition we collected data on a small sample of patients who presented to the ER with fever and were diagnosed with a bacterial infection, such as bacteremia, meningitis, bacterial gastroenteritis, and urinary tract infection (UTI). Bacteremia was defined as a positive blood culture (excluding contaminants: bacillus species [non-anthrax and non-cereus] coagulase negative staphylococcus or corynebacterium sp.). Meningitis was confirmed with positive CSF culture and enteritis with stool culture growth of a bacterial pathogen. Cases of urinary tract infection were diagnosed based on pyuria (>5 WBC on automated urinalysis) and >50,000 CFU of a single uropathogen.

The study was approved by the Institutional Review Board.

## Statistical Analysis

Continuous variables, assumed to be normal, were analyzed using two-sample *t*-test and summarized using mean ± standard deviation. Continuous variables with skewed distribution were analyzed using the Wilcoxon rank-sum test and summarized using median and interquartile range. Categorical variables were summarized using frequency and percent, and analyzed using the Pearson's chi-square test of independence. When the asymptotic assumption of the chi-square distribution was violated, an exact chi-square *p*-value was computed. All analyses were done using SAS 9.4®, and *p*-value < 0.05 were considered significant for all analyses except for the planned comparisons within the viral group where significance was set at 0.025 after Bonferroni correction.

## Results

**Figure 1:**
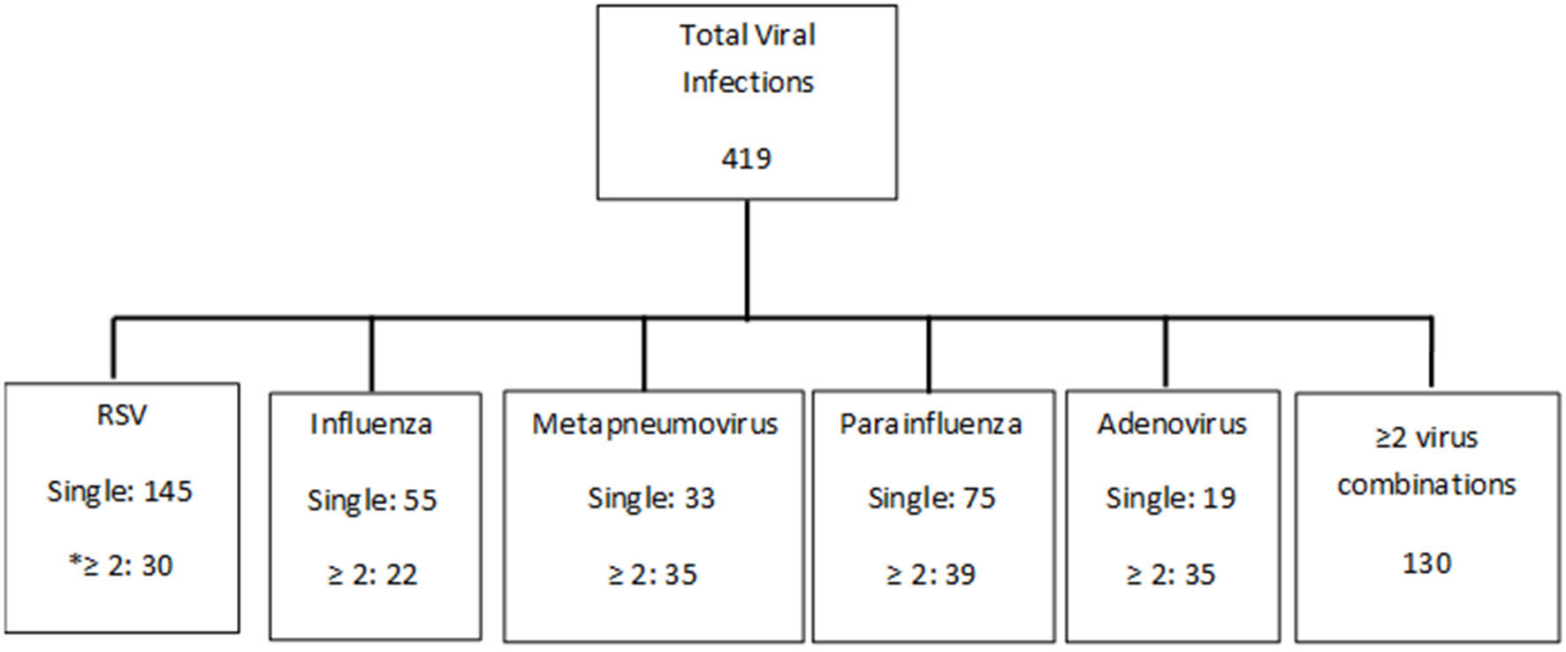
Breakdown of viruses. *≥2 includes of combinations of viruses (most common combination was with enterovirus, rhinovirus, and coronavirus addition to be a combination of the above five viruses).

A total of 419 pediatric patients were included in the study (Figure 1). They all had documented viral infections with no evidence of bacterial coinfection: adenovirus (54), parainfluenza (114), influenza (77), hMPV (68), and RSV (175). Forty five of the cases were combination of the above.

### Viral Group With Band ≥ 10% (Table 1)

In the viral group, patients with band proportions ≥10% had a significantly higher mean absolute neutrophil count (ANC), mean temperature, exposure to antibiotics, and rate of admission than their viral counterpart with band proportions <10%. Furthermore, these patients had significantly lower mean lymphocyte counts and lower mean lymphocyte proportions.

**Table 1:** Bandemia (bands ≥ 10%) in viral infection.

	**Bands <10%**	**Bands ≥ 10%**	**Total**	***P*-value**
Number of participants	362	53	415	
**Demographics**				
Age (months)	16.5 (6.2–29.8)	15.5 (8.3–30.6)	16.0 (6.5–30.5)	0.756
Length of stay (days)	3.0 (2.0–4.0)	2.0 (2.0–5.0)	3.0 (2.0–4.0)	0.683
Duration of fever (days)	2.0 (1.0–4.0)	2.0 (1.0–3.0)	2.0 (1.0–4.0)	0.54
ANC	4219 (2745–7372)	6030 (4046–8909)	4556 (2835–7828)	0.008
Absolute band count	177.0 (0.0–396.0)	1340 (910.0–1780)	225.0 (0.0–591.0)	<0.001
Initial temperature (°C)	100.6 ± 2.1	100.8 ± 2.2	100.6 ± 2.1	0.395
WBC absolute (1,000 K/μL)	11.7 ± 5.6	11.6 ± 5.9	11.7 ± 5.6	0.963
Neutrophil absolute	5.6 ± 4.4	5.4 ± 3.3	5.5 ± 4.3	0.797
Neutrophil proportion	45.2 ± 20.5	45.5 ± 15.1	45.2 ± 19.9	0.92
Lymphocyte absolute	4.8 ± 4.7	3.5 ± 2.1	4.7 ± 4.5	0.04
Lymphocyte proportion	41.7 ± 19.1	31.5 ± 13.8	40.4 ± 18.8	<0.001
Monocyte absolute	1.0 ± 0.7	1.2 ± 1.5	1.0 ± 0.8	0.353
Monocyte proportion	9.1 ± 4.8	8.9 ± 3.6	9.1 ± 4.7	0.807
Female (*N*, %)	185 (51.1%)	24 (45.3%)	209 (50.4%)	0.464
Antibiotics given (*N*, %)	87 (24.0%)	32 (60.4%)	119 (28.7%)	<0.001
Discharge from ED	204 (56.4%)	20 (37.7%)	224 (54.0%)	0.012
Admission to hospital (*N*, %)	158 (43.6%)	34 (64.2%)	192 (46.3%)	0.008
Toxic appearance (*N*, %)	4 (1.1%)	0 (0.0%)	4 (1.0%)	>0.999
Readmission (*N*, %)	7 (1.9%)	1 (1.9%)	8 (1.9%)	>0.999

### RSV Patients With Band ≥ 10% (Table 2)

Patients who were positive for RSV were significantly younger, had lower body temperature, were less likely to receive antibiotics, and were more likely to be admitted to the hospital than those negative for RSV.

**Table 2:** Parameters in individual viruses.

	**Positive**	**Negative**	**Total**	***P*-value**
**RSV**				
Number of participants	175	282	457	
Age (months)	11.6 (4.0–27.0)	17.2 (8.8–31.5)	15.9 (6.2–29.8)	0.001
ANC	4556 (2904–7372)	4612 (2745–8085)	4590 (2835–7828)	0.739
Absolute band count	256.0 (65.0–576.0)	185.5 (0.0–561.0)	218.0 (0.0–561.0)	0.029
Bandemia ≥ 10% (*N*, %)	26 (14.9%)	29 (10.4%)	55 (12.1%)	0.184
Bandemia ≥ 5% (*N*, %)	54 (30.9%)	76 (27.3%)	130 (28.7%)	0.456
Antibiotics given (*N*, %)	42 (24.0%)	89 (31.6%)	131 (28.7%)	0.089
Admission to hospital (*N*, %)	105 (60.0%)	106 (37.6%)	211 (46.2%)	<0.001
**Adenovirus**				
Number of participants	54	403	457	
Age (months)	14.9 (8.6–24.7)	15.9 (5.5–31.0)	15.9 (6.2–29.8)	0.902
ANC	7190 (4864–10465)	4154 (2662–7372)	4590 (2835–7828)	<0.001
Absolute band count	373.0 (77.0–813.0)	208.0 (0.0–489.0)	218.0 (0.0–561.0)	0.046
Antibiotics given (*N*, %)	13 (24.1%)	118 (29.3%)	131 (28.7%)	0.522
Admission to hospital (*N*, %)	15 (27.8%)	196 (48.6%)	211 (46.2%)	0.005
Bandemia ≥ 5% (*N*, %)	18 (35.3%)	112 (27.9%)	130 (28.7%)	0.324
Bandemia ≥ 10% (*N*, %)	9 (17.6%)	46 (11.4%)	55 (12.1%)	0.251
**Influenza**				
Number of participants	77	380	457	
Age (months)	26.3 (13.9–44.1)	14.3 (5.4–26.2)	15.9 (6.2–29.8)	<0.001
ANC	3500 (2312–6156)	4740 (3063–7901)	4590 (2835–7828)	0.004
Absolute band count	159.0 (0.0–395.0)	225.0 (0.0–590.5)	218.0 (0.0–561.0)	0.099
Band proportion	3.2 ± 4.1	3.7 ± 4.4	3.6 ± 4.4	0.373
A/T ratio	0.1 ± 0.1	0.1 ± 0.1	0.1 ± 0.1	0.339
Antibiotics given (*N*, %)	24 (31.2%)	107 (28.2%)	131 (28.7%)	0.583
Admission to hospital (*N*, %)	27 (35.1%)	184 (48.4%)	211 (46.2%)	0.034
Bandemia ≥ 5% (*N*, %)	23 (29.9%)	107 (28.5%)	130 (28.7%)	0.794
Bandemia ≥ 10% (*N*, %)	6 (7.8%)	49 (13.0%)	55 (12.1%)	0.252
**Metapneumovirus**				
Number of participants	68	389	457	
Age (months)	18.4 (9.0–31.1)	15.6 (5.5–29.2)	15.9 (6.2–29.8)	0.28
ANC	4011 (2552–7880)	4608 (2838–7821)	4590 (2835–7828)	0.667
Absolute band count	295.0 (93.5–691.5)	196.0 (0.0–530.0)	218.0 (0.0–561.0)	0.054
Band proportion	4.4 ± 5.0	3.5 ± 4.3	3.6 ± 4.4	0.092
Antibiotics given (*N*, %)	29 (42.6%)	102 (26.2%)	131 (28.7%)	0.009
Admission to hospital (*N*, %)	34 (50.0%)	177 (45.5%)	211 (46.2%)	0.512
Bandemia ≥ 5% (*N*, %)	27 (40.3%)	103 (26.7%)	130 (28.7%)	0.028
Bandemia ≥ 10% (*N*, %)	9 (13.4%)	46 (11.9%)	55 (12.1%)	0.688
**Parainfluenza**				
Number of participants	114	343	457	
Age (months)	15.9 (7.0–24.2)	15.9 (5.8–31.1)	15.9 (6.2–29.8)	0.361
ANC	4224 (2520–6966)	4615 (2916–7917)	4590 (2835–7828)	0.182
Absolute band count	132.5 (0.0–345.0)	252.0 (0.0–681.0)	218.0 (0.0–561.0)	<0.001
Band proportion	2.4 ± 3.5	4.0 ± 4.6	3.6 ± 4.4	0.001
A/T ratio	0.1 ± 0.1	0.1 ± 0.1	0.1 ± 0.1	0.008
Antibiotics given (*N*, %)	32 (28.1%)	99 (28.9%)	131 (28.7%)	0.905
Admission to hospital (*N*, %)	45 (39.5%)	166 (48.4%)	211 (46.2%)	0.105
Bandemia ≥ 5% (*N*, %)	18 (15.8%)	112 (33.0%)	130 (28.7%)	<0.001
Bandemia ≥ 10% (*N*, %)	9 (7.9%)	46 (13.6%)	55 (12.1%)	0.135

### Adenovirus Patients With Band ≥ 10%

Patients who were positive for adenovirus had higher ANC, body temperature, WBC, absolute neutrophil counts, and absolute monocyte counts. In addition, they were less likely to be admitted to the hospital than patients negative for adenovirus.

### Parainfluenza Patients With Band ≥ 10%

Patients who were positive for parainfluenza virus had lower rates of admission to the hospital, and lower rate of patients with bandemia ≥ 10% when compared to patients negative for parainfluenza. Of note, these patients had higher lymphocyte proportions.

### Influenza Patients With Band ≥ 10%

Patients who were positive for influenza were older and had lower WBC, absolute lymphocyte counts, and absolute monocyte counts than patients negative for influenza.

### Metapneumovirus Patients With Band ≥ 10%

Patients who were positive for hMPV had higher band proportions and exposure to antibiotics when compared to patients negative for hMPV.

### Combination Group With Band ≥ 10%

Patients who were positive for 2 or more viruses had higher ANC, temperature, and WBC but had shorter length of stay in the hospital when compared to those positive with only one virus.

### Bacterial vs. Viral Group

In our sample of 42 patients diagnosed with bacterial infections, there were urinary tract infections (36), Streptococcus pneumonia meningitis (2), bacteremia Escherichia coli (2), and Shigella enteritis (2).

We compared characteristics between the bacterial group and the viral group. Patients with a bacterial infection were significantly younger than patients with a viral infection. Patients with a bacterial infection had significantly higher mean ANC, mean WBC, mean neutrophil count, and higher exposure to antibiotics than their viral counterparts (Table 3). When comparing the absolute band count and band proportion between the viral group and the bacterial group, there was no statistically significant difference. When comparing the WBC and ANC between the viral group and the bacterial group, the bacterial group had a higher WBC and ANC which was significant (*p* <0.001).

**Table 3:** Demographic and clinical characteristic.

	**Viral**	**Bacterial**	**Total**	***P*-value**
Number of participants	419	42	461	
**Demographics**
Age (months)	15.9 (6.5–30.5)	7.1 (4.0–26.7)	15.6 (6.0–29.2)	0.008
Length of stay (days)	3.0 (2.0–4.0)	3.0 (3.0–4.0)	3.0 (2.0–4.0)	0.128
Duration of fever (days)	2.0 (1.0–4.0)	1.0 (1.0–3.0)	2.0 (1.0–3.0)	0.102
ANC	4558 (2835–7828)	8638 (4950–11700)	4717 (2904–8076)	<0.001
Absolute band count	222.0 (0.0–590.0)	248.5 (0.0–868.0)	225.0 (0.0–648.0)	0.275
Initial temperature (°C)	100.6 ± 2.1	100.6 ± 2.4	100.6 ± 2.1	0.974
WBC absolute (weeks)	11.7 ± 5.6	16.8 ± 7.5	12.1 ± 6.0	<0.001
Band proportion	3.7 ± 4.5	3.8 ± 4.8	3.7 ± 4.5	0.885
Neutrophil absolute	5.5 ± 4.3	7.4 ± 4.1	5.7 ± 4.3	0.009
Neutrophil proportion	45.3 ± 19.8	48.5 ± 18.4	45.6 ± 19.7	0.319
Lymphocyte absolute	4.7 ± 4.5	5.7 ± 4.1	4.8 ± 4.5	0.145
Lymphocyte proportion	40.3 ± 18.8	36.9 ± 16.2	40.0 ± 18.5	0.251
Monocyte absolute	1.0 ± 0.8	1.2 ± 1.0	1.1 ± 0.8	0.491
Monocyte proportion	9.1 ± 4.7	6.8 ± 5.3	8.9 ± 4.8	0.004
A/T ratio	0.1 ± 0.1	0.1 ± 0.1	0.1 ± 0.1	0.861
Female (*N*, %)	211 (50.4%)	25 (59.5%)	236 (51.2%)	0.331
Antibiotics given (*N*, %)	119 (28.4%)	39 (92.9%)	158 (34.3%)	<0.001
Admission to hospital (*N*, %)	192 (45.8%)	26 (61.9%)	218 (47.3%)	0.052
Toxic appearance (*N*, %)	4 (1.0%)	0 (0.0%)	4 (0.9%)	>0.999
Readmission (*N*, %)	9 (2.1%)	0 (0.0%)	9 (2.0%)	>0.999

## Discussion

**Figure 2:**
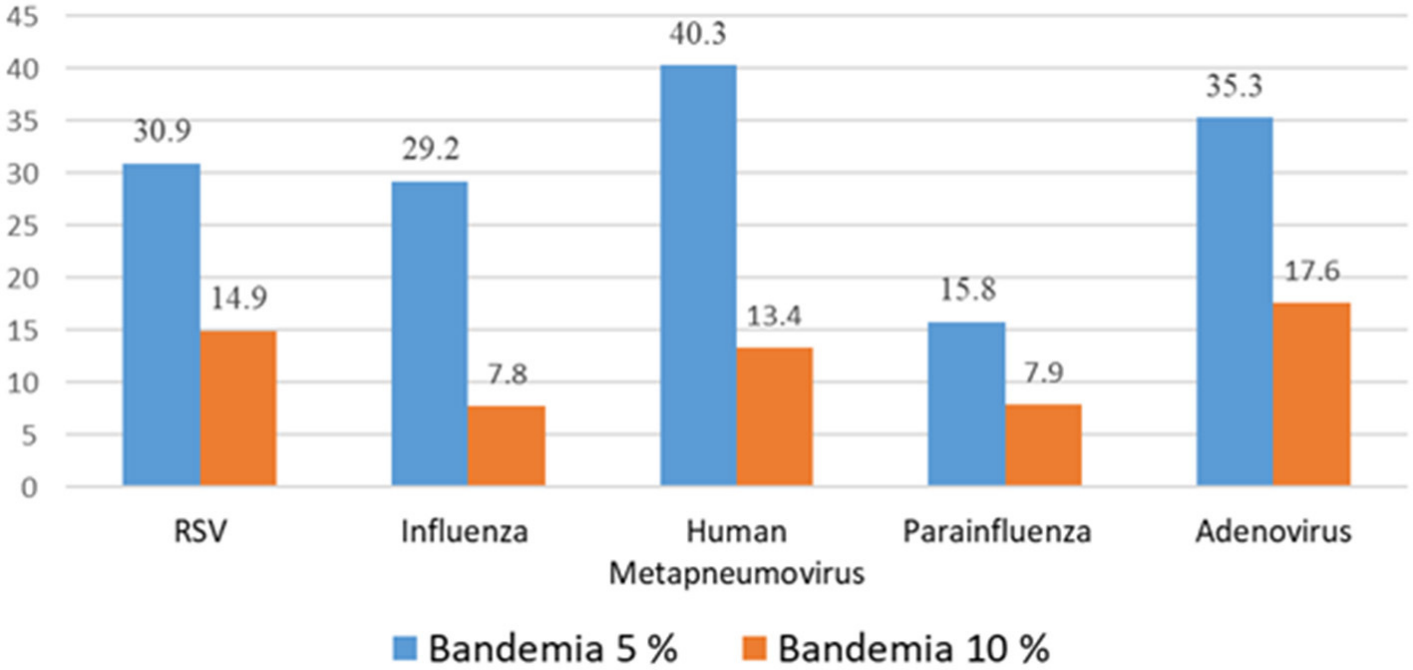
% Bandemia in Respiratory viral Infections.

Bandemia or elevated immature neutrophil count (left shift) was detected in a cohort of febrile children presenting to the emergency department with confirmed respiratory viral infection without a concomitant bacterial infection. In our study, a significant proportion of these children were found to have bandemia. Adenovirus (17%), RSV (14.9%), human metapneumovirus (13%), and parainfluenza (7.9%) had the highest prevalence when the cutoff for bandemia was set at 10%. The prevalence increased to 35.3, 30.9, 40.3, and 15.8% for adenovirus, RSV, hMPV, and PIV, respectively when this cutoff was lowered further to 5%, which is the cutoff used by our institution (Figure 2).

Over the past decade, use of multiplex molecular respiratory panel has enabled better identification of respiratory viral infections. As a result, this information has allowed us to investigate the differential white blood cell count in common childhood viral infections (8). We reviewed the different cell lines, including immature neutrophils (bands), lymphocytes, and monocytes. We found that adenovirus had significantly (*p* <0.001) higher ANC when compared to the other viruses with a mean value of 7,190.

Although identification of bandemia may represent a serious underlying bacterial infection (SBI) (9, 10) immature neutrophils are also associated with certain viral infections (11). Of note, in our study, we found viral cases with band proportions >10% which had significantly higher exposure to antibiotics and hospital admission rates (*p* <0.001). The study results suggest that the traditional belief of bandemia and its association with SBI may persist in clinical practice.

**Figure 3:**
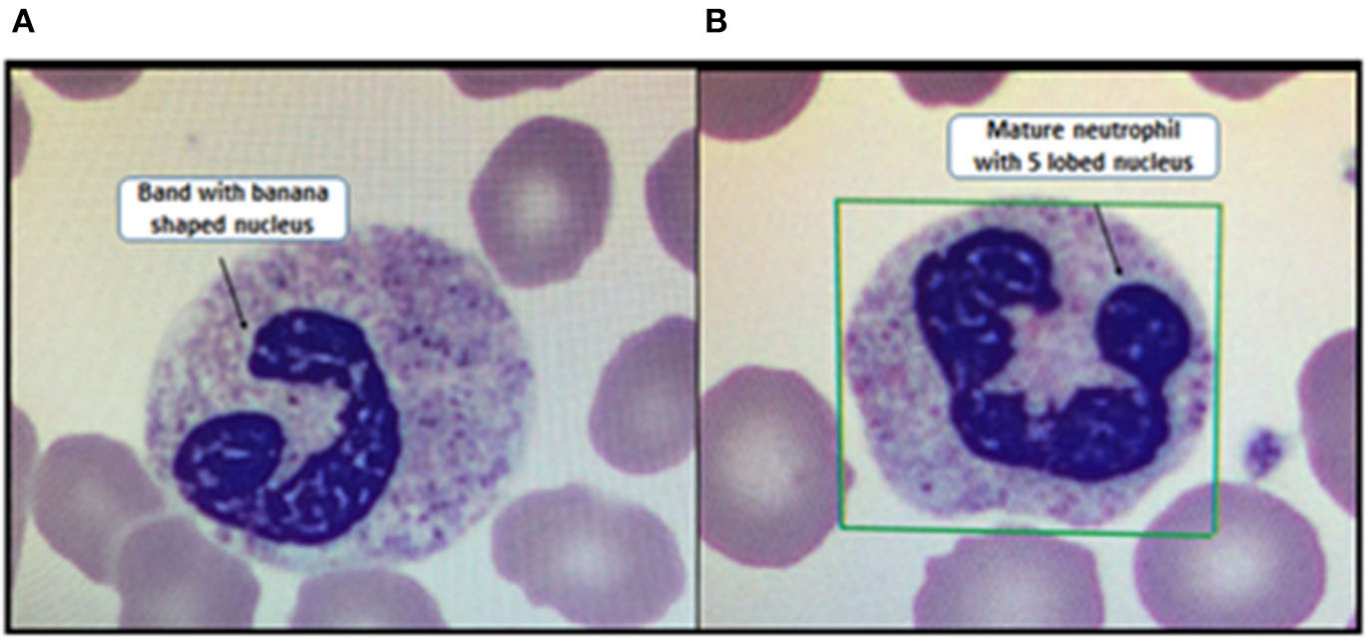
**(A)** Immature neutrophil vs. **(B)** mature neutrophil.

*What is bandemia?* WBC is a component of the CBC. The WBC's differential measures the absolute number and proportion of granulocytes, lymphocytes, monocytes, basophils, and eosinophils (12). A neutrophil is a mature granulocyte and part of the innate immune system. Band is the most “mature” of the immature stages in the development of a neutrophil (Figure 3). In older children and adults, the normal blood circulation has 3–5% of bands which will mature into neutrophils in the circulation. However, the majority of blasts undergo maturation in the marrow storage compartment. Any stress, whether an infection, inflammation, or a hypermetabolic state will lead to an increase in band count or bandemia (>10%) (13–15).

In our study, all differentials were performed manually. Manual band count requires the additional time-consuming, labor-intensive process of slide preparation and manual leukocyte tabulation. The interpretation is technician dependent, and its enumeration is associated with inaccuracy and imprecision. In addition, there exists a wide variation in reference ranges of bandemia (16). It has been reported in the literature that the percentage and absolute band count in healthy children between the ages of 15 days and 2 years of age is similar, but the response to infection may be different in young children of different ages (16).

*What does presence of bandemia in viral infections mean?* Bandemia has been reported in children with viral infections notably with lower respiratory tract infections, enteroviral infections, and rotavirus infections (6, 11, 17). Bandemia has also been reported in association with influenza and RSV infections (18).

We found bandemia (>10%) in about a quarter of cases of respiratory viral infections in the absence of concomitant bacterial infection (confirmed or presumed): RSV (14.9% 26/175), adenovirus (17.6% 9/54), hMPV (13.4% 9/68), influenza (7.8% 6/77), and parainfluenza virus (7.9% 9/114). The cutoff for bandemia in our laboratory is 5% which further increases the percentage of children classified with bandemia. This implies that a relatively higher band count is seen in viral infections that primarily cause lower respiratory tract disease and are associated with more parenchymal inflammation at the time of ER presentation. In addition, our study is the first to report the prevalence of bandemia in hMPV infections.

In reviewing our data we noticed that patients admitted with a known viral infection, but high band count, were likely to receive antibiotics. Antibiotic exposure was seen in one quarter of the patients infected with hMPV (40.3%), followed by influenza (31%), parainfluenza (28%), RSV (24%), and adenovirus (17.6%). This lower antibiotic use in adenovirus can be attributed to the known association of bandemia in adenovirus by clinicians (Table 4).

**Table 4:** Bandemia in specific viruses with antibiotic prescription and hospitalization percent.

**Viral**	**Band**	**Band**	**Mean absolute**	**Antibiotic**	**Hospitalization**
**group**	**> 5%**	**> 10%**	**band count**	**% (*n*)**	**% (*n*)**
RSV	31%	15%	256	24% (42)	60 % (105)
Adenovirus	35%	17.6%	373	24% (13)	28 % (15)
Influenza	30%	7.8%	159	31 % (24)	35 % (27)
Parainfluenza	16%	7.9%	132	28 % (32)	39 % (45)
Metapneumovirus	40.3%	13%	295	42.6 % (29)	50 % (34)

We compared the bacterial group with the viral group and did not find a statistically significant difference in bandemia when comparing viral with bacterial infections in infants and young children. However, the bacterial group was a considerably smaller sample size so when comparing the groups, the results should be taken cautiously. Nevertheless, when comparing the absolute band count, band proportion, and A/T ratio between the viral group and the bacterial group, there was no statistical significant difference.

This study has several limitations. First, it is a retrospective review conducted in a single center. Second, there were fewer patients in the bacterial group in comparison to the viral group; therefore, the incidence of true bandemia in the bacterial group might be under represented. In addition, we included only well-appearing children with confirmed viral infections, thus we cannot generalize our results to ill appearing patients.

## Conclusions

Elevated band counts are seen in lower respiratory viral infections and clinicians should be aware of this association. While absolute neutrophil count and white blood cell count are reliable markers of bacterial infection, bandemia may also be present in viral infections. This is the first study comparing bandemia among different viral infections. We observed an elevated band count in many viral infections, specifically RSV, adenovirus, hMPV, influenza, and parainfluenza. Bandemia in well-appearing children with confirmed viral infections can be an area of opportunity to limit the use of antibiotics.

## Data Availability Statement

The raw data supporting the conclusions of this article will be made available by the authors, without undue reservation.

## Ethics Statement

The studies involving human participants were reviewed and approved by NYU Long Island School of Medicine IRB Department. Written informed consent from the participants' legal guardian/next of kin was not required to participate in this study in accordance with the national legislation and the institutional requirements.

## Author Contributions

EN, AN, and RR was involved in manuscript writing and data collection of viral infections. JC was involved in data collection of bacterial infections. RC performed statistical analysis. NS was involved in manuscript writing, and LK supervised the project and was involved in revision of the manuscript. All authors contributed to the article and approved the submitted version.

## Conflict of Interest

The authors declare that the research was conducted in the absence of any commercial or financial relationships that could be construed as a potential conflict of interest.

## Abbreviations

CBC: complete blood count
WBC: white blood cell count
ANC: absolute neutrophil count
RSV: Respiratory syncytial virus
hMPV: human metapneumovirus
ER: emergency room
SBI: serious bacterial infection
PCR: polymerase chain reaction.
